# Co-located 18S/5S rDNA arrays: an ancient and unusual chromosomal trait in Julidini species (Labridae, Perciformes)

**DOI:** 10.3897/CompCytogen.v10i4.10227

**Published:** 2016-11-04

**Authors:** Karlla Danielle Jorge Amorim, Marcelo de Bello Cioffi, Luiz Antonio Carlos Bertollo, Rodrigo Xavier Soares, Allyson Santos de Souza, Gideão Wagner Werneck Felix da Costa, Wagner Franco Molina

**Affiliations:** 1 Departamento de Biologia Celular e Genética, Centro de Biociências, Universidade Federal do Rio Grande do Norte, Campus Universitário, Lagoa Nova, 3000, 59078-970, Natal, RN, Brasil; 2 Departamento de Genética e Evolução, Universidade Federal de São Carlos, Rodovia Washington Luís, km 235,13565-905, São Carlos, SP, Brasil

**Keywords:** Chromosome evolution, Halichoeres, rDNA, syntenic genes, wrasses

## Abstract

Wrasses (Labridae) are extremely diversified marine fishes, whose species exhibit complex interactions with the reef environment. They are widely distributed in the Indian, Pacific and Atlantic oceans. Their species have displayed a number of karyotypic divergent processes, including chromosomal regions with complex structural organization. Current cytogenetic information for this family is phylogenetically and geographically limited and mainly based on conventional cytogenetic techniques. Here, the distribution patterns of heterochromatin, GC-specific chromosome regions and Ag-NORs, and the organization of 18S and 5S rDNA sites of the Atlantic species *Thalassoma noronhanum* (Boulenger, 1890), *Halichoeres poeyi* (Steindachner, 1867), *Halichoeres radiatus* (Linnaeus, 1758), *Halichoeres brasiliensis* (Bloch, 1791) and *Halichoeres penrosei* Starks, 1913, belonging to the tribe Julidini were analyzed. All the species exhibited 2n=48 chromosomes with variation in the number of chromosome arms among genera. *Thalassoma noronhanum* has 2m+46a, while species of the genus *Halichoeres* Rüppell, 1835 share karyotypes with 48 acrocentric chromosomes. The *Halichoeres* species exhibit differences in the heterochromatin distribution patterns and in the number and distribution of 18S and 5S rDNA sites. The occurrence of 18S/5S rDNA syntenic arrangements in all the species indicates a functionally stable and adaptive genomic organization. The phylogenetic sharing of this rDNA organization highlights a marked and unusual chromosomal singularity inside the family Labridae.

## Introduction

Wrasses (Labridae) are one of the most abundant and ecologically diversified fish groups in tropical reefs (Choat and Bellwood 1998). Their biodiversity is highlighted by nine tribes with 82 genera and 530 species (Westneat and Afaro 2005, Eschmeyer and Fong 2016), which exhibit extensive biological interactions in tropical reefs and temperate regions around the world (Choat and Bellwood 1998, Wainwright et al. 2004, Nelson 2006).

Cytogenetic analyses in Labridae have revealed particular trends in the karyotypic evolution of their clades (Sena and Molina 2007). In fact, pericentric inversions stand out as the major chromosomal rearrangements in the evolution of the tribes Hypsigenyini, Scarini and Julidini (Sena and Molina 2007, Molina et al. 2012). In turn, in the Novaculini, Cheilini, Pseudocheilini and Labrini tribes, both pericentric inversions and chromosome fusions have contributed for their karyotypic diversification (Ueno and Takai 2000).

In general, Labridae clades can be differentiated into four karyotypic patterns. The first one is characterized by conserved karyotypes, with 48 acrocentric chromosomes; the second by 48 chromosomes with an increase in the chromosome arms (NF); the third by a reduction in the number of chromosomes (<48 chromosomes) but with the same NF; and the fourth by reduced diploid number and NF (Alvarez et al. 1986, Sena and Molina 2007).

At the moment, Julidini is the clade with the largest amount of cytogenetic data in Labridae (Table 1). Nonetheless, they are based on conventional cytogenetic methods and very incipient yet, given its species’ diversity. This tribe falls mainly into the first and second karyotypic patterns, with conserved diploid values (2n=48), mostly acrocentric chromosomes, or with variations in the NF due to pericentric inversions. Different classes of repetitive DNAs are linked to chromosome rearrangements in many fish groups (Kidwell 2002, Cioffi and Bertollo 2012, Getlekha et al. 2016). Indeed, repetitive DNAs may clarify the occurrence of particular chromosome rearrangements and evolutionary relationships among different taxa (Shapiro and Sternberg 2005, Biémont and Vieira 2006, Artoni et al. 2015). However, the chromosome organization and the evolutionary dynamics of this important fraction of the genome are still poorly understood in Labridae fishes.

**Table 1.** Variations in diploid values (2n) and number of chromosome arms (NF) among Labridae fishes (adapted from Sena and Molina 2007, Arai 2011).

Tribe	N	2n range/ Modal value	NF range/ Modal value	NF Average
Hypsigenyini	7	48/48	56–86/78	76
Pseudocheilini	8	34–48/34	46–84/46	65
Julidini	32	48/48	48–86/48	52
Labrini	10	38–48/48	48–86/48	46
Scarini	5	46–48/48	66–88/66	74
Cheilini	8	32–48/48	38–84/60	66
Labrichthyines	1	48	48	48
Novaculini	8	22–48/48	40–56/48	47
Pseudolabrini	1	48	52	52

Among Julidini wrasses, *Halichoeres* Rüppell, 1835 is the most diversified and polyphyletic genus, comprising distinct components in the New World and Indo–Pacific Ocean (Barber and Bellwood 2005, Westneat and Alfaro 2005, Rocha et al. 2010). *Thalassoma* Swainson, 1839, phylogenetically close to *Halichoeres*, dates from 8–13 mya and contains 27 species, with a marked increase in diversification between 5–10 mya (Bernardi et al. 2004). *Thalassoma noronhanum* (Boulenger, 1890) is one of the smallest known species (Allen 1995), with a wide occurrence on the Brazilian coast and a number of oceanic islands in the Western Atlantic. Despite some cytogenetic data available for *Halichoeres* species (Sena and Molina 2007), there are no information for *Thalassoma* ones from the Atlantic (Arai 2011). In the present study, cytogenetic investigation on C-banding, Ag-NORs, base-specific fluorochrome staining and double-fluorescence *in situ* hybridization (FISH) with 18S rDNA and 5S rDNA probes, were realized in five Julidini species. The data were useful to clarify particular chromosomal processes and phylogenetic relationships of these marine fish species, besides evidencing an unusual co-localization of 18S and 5S rDNA clusters in all species.

## Material and methods

### Specimens and chromosomal preparation

The specimens of *Halichoeres poeyi* (Steindachner, 1867) (N=13) and *Halichoeres brasiliensis* (Bloch, 1791) (N=6) were collected in the coast of Rio Grande do Norte (5°42'20"S, 35°11'38"W), Northeastern Brazil. Individuals of *Halichoeres radiatus* (Linnaeus, 1758) (N=16) were obtained from the Fernando de Noronha Archipelago (3°51'20"S, 32°25'32"W), *Halichoeres penrosei* Starks, 1913 (N=3) from the Trindade Island (20°30'13"S, 29°19'50"W) and *Thalassoma noronhanum* from the Rocas Atoll (N=22) (3°51'59"S, 33°48'20"W).

The specimens were submitted to intraperitoneal mitotic stimulation with fungal and bacterial antigen complexes (Molina et al. 2010). Mitotic chromosome preparations were obtained by *in vitro* methodology, using a cell suspension of kidney tissue fragments (Gold et al. 1990). The C-positive heterochromatin and nucleolus organizer regions (NORs) were visualized using the C-banding and Ag-NOR staining (Sumner 1972, Howell and Black 1980, respectively). Chromosomes were also stained with mithramycin (MM) and 4’, 6-diamidino-2-phenylindole (DAPI) fluorochromes, according to Schweizer (1976).

### Obtaining probes for chromosomal hybridization

The 5S and 18S rDNA probes, containing approximately 200 pb and 1400 pb, respectively, were obtained by polymerase chain reaction (PCR) from the nuclear DNA of *Rachycentron canadum*, using the primers A 5’-TAC GCC CGA TCT CGT CCG ATC-3’ and B 5’- CAG GCT GGT ATG GCC GTA AGC-3’ (Pendás et al. 1994), NS1 5’-GTA GTC ATA TGC TTG TCT C-3’ and NS8 5’-TCC GGT GCA TCA CCT ACG GA-3’ (White et al. 1990), respectively. The 18S rDNA and 5S rDNA probes were labeled with digoxigenin-11-dUTP (Roche, Mannheim, Germany) and biotin-14-dATP (Invitrogen^TM^), respectively, according to manufacturer’s specifications.

### Chromosomal hybridization


 Fluorescence *in situ* hybridization (FISH) was performed according to Pinkel et al. (1986). Slides with metaphase chromosomes were first treated with RNAse (20 µg/ml in 2XSSC) at 37°C for 1 hour and with pepsin (0.005% in 10mM HCl), for 10 minutes, fixed with 1% formaldehyde for 10 minutes and dehydrated in alcohol baths (70%/85%/100%) for 5 minutes each. The chromosomes were then incubated in 70% formamide/2XSSC at 72°C, for 5 minutes and once again dehydrated in an alcohol series (70%/85%/100%). The hybridization was performed at 37°C for 16h, using a hybridization solution consisting of 50% formamide, 2XSSC, 10% dextran sulfate and the denatured probe (5 ng/µl), with a final volume of 30 µl. Post-hybridization washings were done in 15% formamide/0.2XSSC at 42°C, for 20 minutes, followed by washings in 0.1XSSC at 60°C for 15 minutes and in Tween 20 (0.5%/4XSSC) for 5 minutes, at 25°C. Next, the slides were incubated for 15 minutes in a blocking solution (5% NFDM /4xSSC) and washed with Tween 20 (0.5%/4XSSC) for 15 minutes. The hybridization signals were detected using anti-digoxigenin rhodamine (Roche, Mannheim, Germany) for the 18S rDNA probe and streptavidin-FITC (Vector Laboratories) for the 5S rDNA probe. The chromosomes were counterstained with Vectashield/DAPI (1.5 µg/ml) (Vector).

At least thirty metaphase spreads were analyzed to confirm the diploid chromosome numbers, karyotype structure and FISH results. The best metaphases were photographed using an Olympus^TM^ BX50 epifluorescence microscope, coupled to an Olympus DP73 digital capture system. The chromosomes were classified as submetacentric (sm) and acrocentric (a), according to the arm ratio (Levan et al. 1964), and arranged in decreasing order of size in the karyotypes.

## Results

All species showed a high number of acrocentric chromosomes. *Thalassoma noronhanum* has 2n=48, with 2sm+46a (NF=50) (Fig. 2a). The species *Halichoeres radiatus*, *Halichoeres poeyi*, *Halichoeres brasiliensis* and *Halichoeres penrosei* have symmetric karyotypes, with 2n=48 acrocentric chromosomes (NF=48) (Fig. 2b-e). The heterochromatin occupies the centromeric and pericentromeric regions of all chromosomes, and also the telomeric regions of a few (Fig. 2a–e).

**Figure 1.**
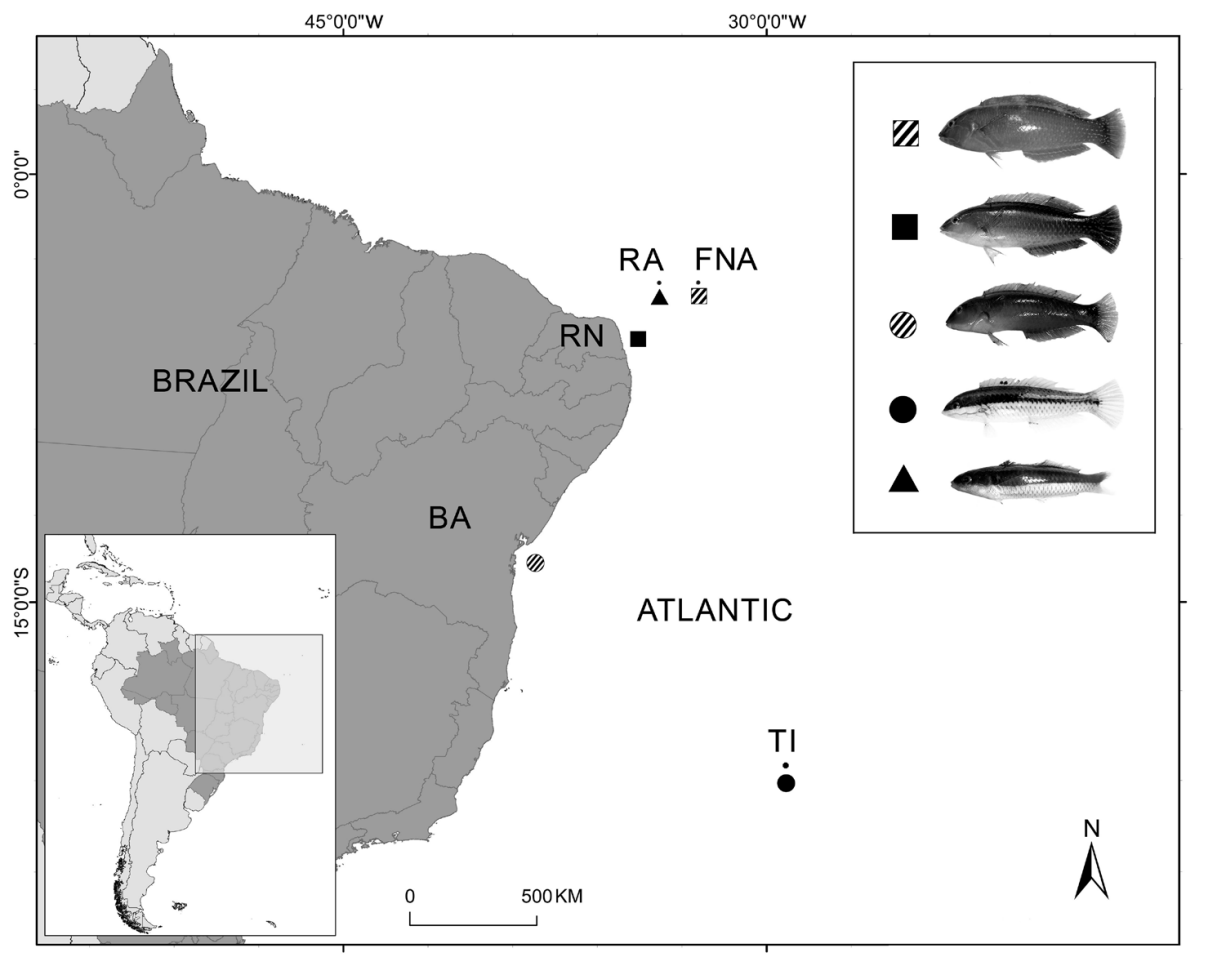
Collection points of the Labridae species analyzed. Fernando de Noronha Archipelago (FNA); Rocas Atoll (RA); Rio Grande do Norte coast (RN); Bahia coast (BA); and Trindade Island (TI). *Halichoeres radiatus* (FNA), *Halichoeres brasiliensis* (RN), *Halichoeres poeyi* (BA), *Halichoeres penrosei* (TI) and *Thalassoma noronhanum* (RA).

**Figure 2.**
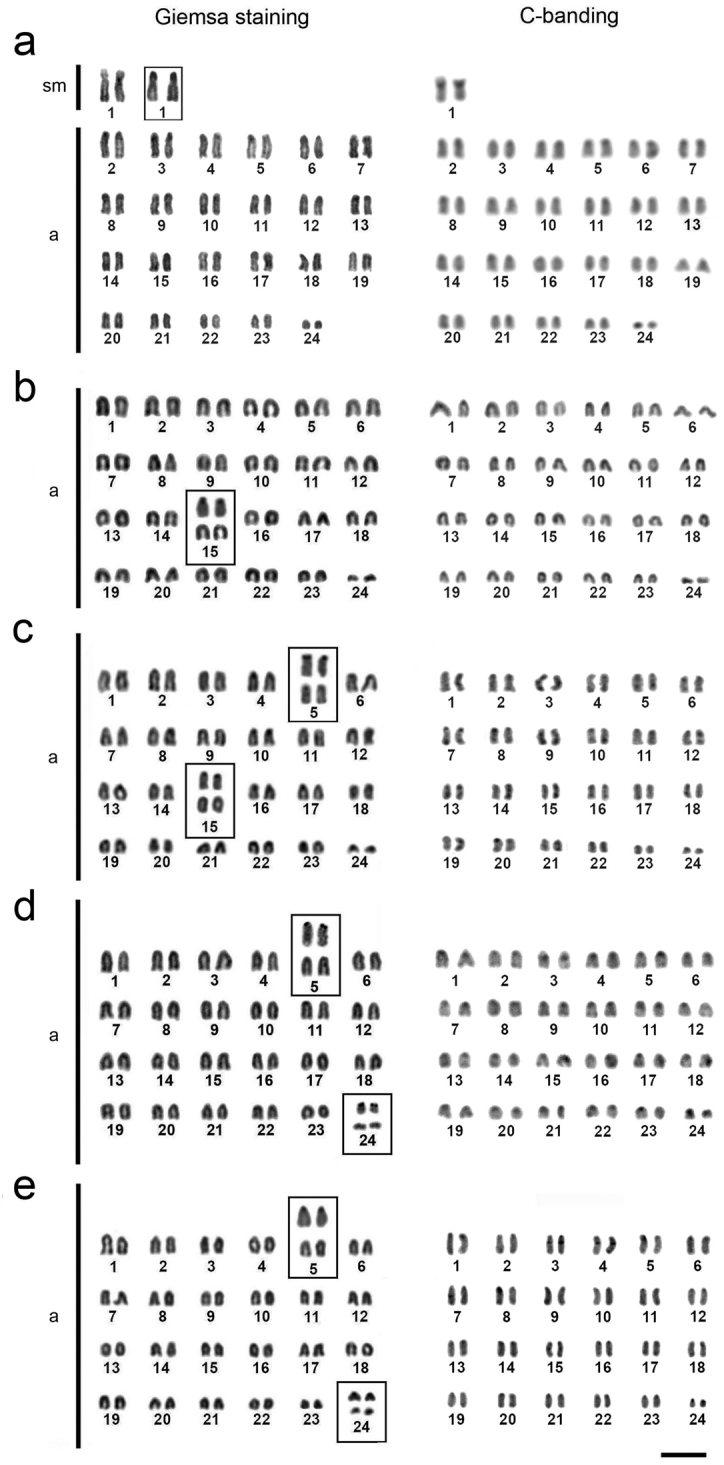
Karyotypes of *Thalassoma noronhanum* (**a**), *Halichoeres penrosei* (**b**), *Halichoeres poeyi* (**c**), *Halichoeres radiatus* (**d**), and *Halichoeres brasiliensis* (**e**). The chromosomal pairs bearing Ag-NORs are boxed, the silver staining in the upper row. Bar: 5 μm.

The Ag-NORs are positioned on the short arms of the single submetacentric pair of *Thalassoma noronhanum* (Fig. 2a). In *Halichoeres* species, these sites are located in two chromosome pairs, except in *Halichoeres penrosei*, where they are located on the short arms of pair 15 (Fig. 2b, highlighted). In *Halichoeres poeyi*, the Ag-NORs occupy the short arms of pairs 5 and 15 (Fig. 2c) and in *Halichoeres radiatus* and *Halichoeres brasiliensis* the short arms of pairs 5 and 24 (Fig. 2d, e).

**Figure 3.**
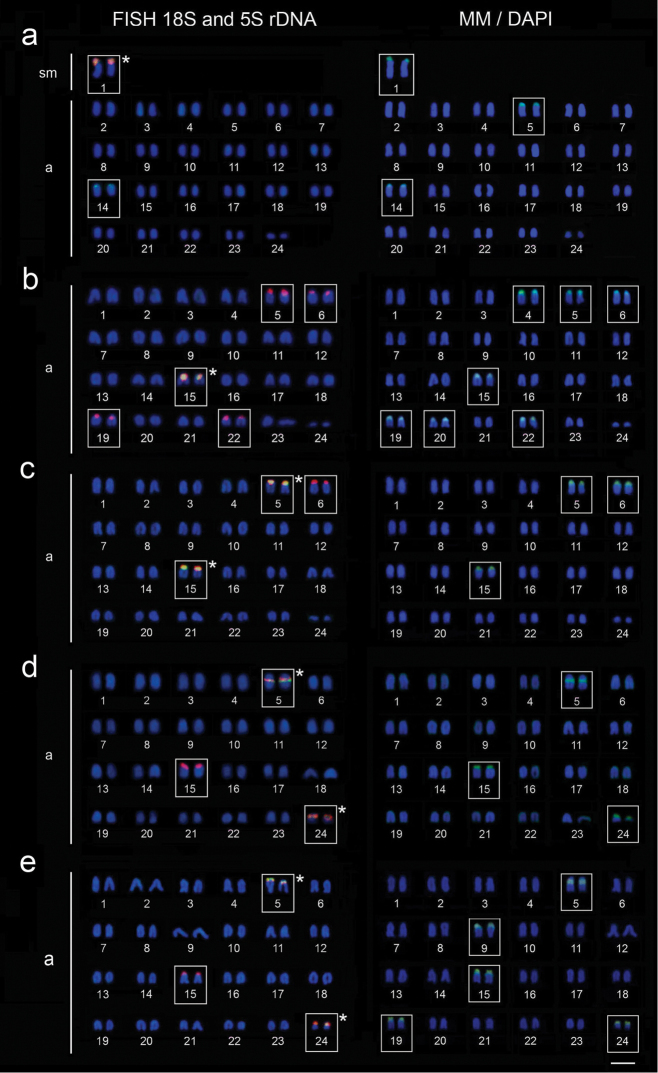
Double-FISH with18S rDNA (red) and 5S rDNA (green) probes and MM/DAPI fluorochromes staining in the chromosomes of *Thalassoma noronhanum* (**a**), *Halichoeres penrosei* (**b**), *Halichoeres poeyi* (**c**), *Halichoeres radiatus* (**d**) and *Halichoeres brasiliensis* (**e**). Asterisks indicate the chromosome pairs with 18S/5S rDNA arrays. Bar: 5 μm.

The mapping of 18S rDNA sequences showed single sites in *Thalassoma noronhanum*, coincident with the Ag-NORs (Fig. 3a). On the contrary, all *Halichoeres* species have multiple 18S rDNA sites. They occur in the terminal position of the short arms of pairs 5, 6, 15, 19 and 22 in *Halichoeres penrosei* (Fig. 3b), of pairs 5, 6 and 15 in *Halichoeres poeyi* (Fig. 3c), and of pairs 5, 15 and 24 of *Halichoeres brasiliensis* (Fig. 3e). However, in *Halichoeres radiatus* they are found in interstitial position on pair 5 and in terminal position on pairs 15 and 24 (Fig. 3d).

The 5S rDNA sites occur in an 18S/5S rDNA array in pair 1 and exclusively on the short arms of pair 14 in *Thalassoma noronhanum* (Fig. 3a). On the other hand, in all *Halichoeres* species, the 5S rDNA sites are co-located with the 18S rDNA ones. They occur in the terminal position of the short arms of pair 15 in *Halichoeres penrosei* (Fig. 3b), of pairs 5 and 15 in *Halichoeres poeyi* (Fig. 3c), of pairs 5 and 24 in *Halichoeres brasiliensis* (Fig. 3e). In *Halichoeres radiatus* they are found in interstitial position in pair 5 and terminal position in pair 24 (Fig. 3d). The Ag-NOR marks were located exclusively on the 18S/5S rDNA arrays.

The sequential staining with MM/DAPI fluorochromes showed a larger number of GC-rich regions than rDNA sites in *Thalassoma noronhanum*, *Halichoeres penrosei* and *Halichoeres brasiliensis*. However, in all the species, the 18S and 5S rDNA sites and 18S/5S rDNA arrays were coincident with GC-rich regions (Fig. 3a–e).

## Discussion

The rates of chromosome diversification can vary significantly among marine fish families (Molina et al. 2014) and, in some cases, they are linked to the evolutionary dynamics of the rDNA sequences. Indeed, groups with marked karyotype conservatism (Molina 2007) usually exhibit low diversification in the frequency and organization of ribosomal sites (Motta-Neto et al. 2011, Calado et al. 2013), while those with moderate or higher rates of chromosomal diversification (Molina et al. 2014) may display marked variations in the rDNA regions (Lima-Filho et al. 2014a, b).

In contrast with several Perciformes groups, Labridae show considerable variation in the diploid values (2n=22 to 48), as well as in the number of chromosome arms (NF=38 to 92) (Sena and Molina 2007, Arai 2011). The evolutionary rates of chromosomes differ significantly among clades (Table 1), reflecting their different histories linked to a deep association with coral reefs (Wainwright et al. 2004).

The cytogenetic patterns of the five analyzed wrasses suggest a greater karyotype conservatism in Julidini than in other Labridae clades. Indeed, *Halichoeres* and *Thalassoma* species exhibit karyotypes with 2n=48 chromosomes, mostly or entirely formed by acrocentric chromosomes, small amount of heterochromatin and one or two pairs bearing Ag-NORs (Sena and Molina 2007, present paper), a characteristic recognized as basal for Perciformes (Brum and Galetti 1997, Galetti et al. 2000).

The chromosomal divergences in Julidini are mainly due to a small number of pericentric inversions (Table 1). In *Thalassoma noronhanum*, the presence of an exclusive pair of biarmed chromosomes demonstrates a variant condition with respect to six other species previously described in this genus, all of them with 2n=48a. On the other hand, in *Halichoeres* species the presence of few biarmed chromosomes (1 to 3 pairs) is relatively more frequent (Sena and Molina 2007, Arai 2011), albeit not identified in the Atlantic species here investigated. However, despite the similarities in the karyotype structure of *Thalassoma noronhanum*, *Halichoeres penrosei*, *Halichoeres poeyi*, *Halichoeres radiatus* and *Halichoeres brasiliensis*, a dynamic evolutionary condition concerning the rDNA regions occurs among these species, which contribute to understanding the karyotypic evolution in Julidini. In fact, the chromosome mapping of rDNA sequences showed a significant variation in frequency, distribution and organization, especially in the *Halichoeres* species.

Chromosomes with homogeneous and small amounts of repetitive DNAs have been found in fish species with little karyotype diversification (Molina 2007, Motta-Neto et al. 2011). On the other hand, heterogeneous and large amounts of repetitive DNAs are related in several families with notable levels of chromosomal rearrangements and differentiation (Moreira-Filho and Bertollo 1991, Souza et al. 2001, Favarato et al. 2016). Among the repetitive DNAs, rDNA has a major role in karyotype diversification. In fact, species from various fish families exhibit 18S and 5S rDNAs sequences involved in chromosome fusion points (Molina and Galetti 2002, Ziemniczak 2011, Jacobina et al. 2013, Getlekha et al. 2016), indicating their probable involvement in the chromosomal reorganization. In this sense, the presence of an 18S rDNA site in the interstitial position on pair 5 in *Halichoeres radiatus*, in contrast to its terminal position in the homeologous chromosomes of the remaining species, puts in evidence a cryptic paracentric inversion in that chromosome pair.

**Figure 4.**
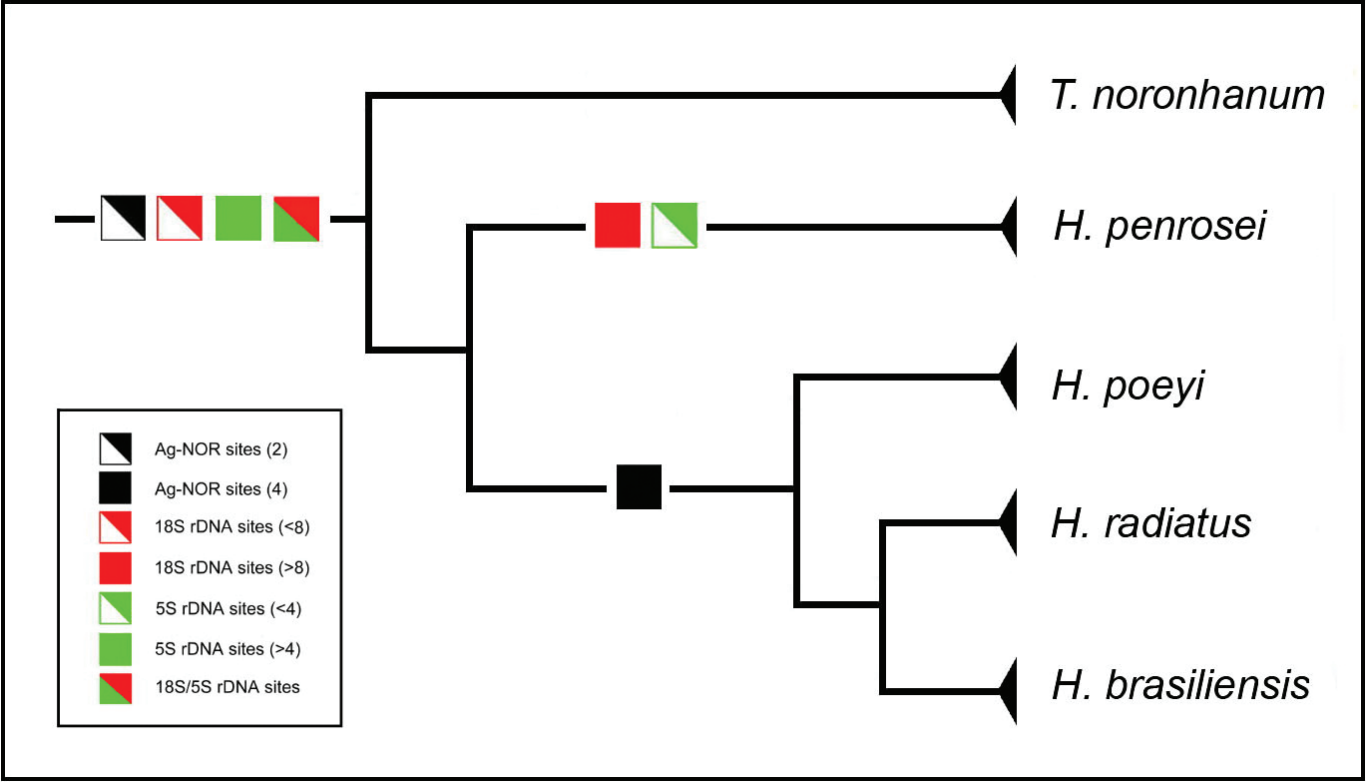
Evolutionary patterns of ribosomal sites in *Thalassoma noronhanum*, *Halichoeres radiatus*, *Halichoeres poeyi*, *Halichoeres brasiliensis* and *Halichoeres penrosei*, from the phylogenetic perspective (evolutionary relationships adapted from Rocha et al. 2010).

From the phylogenetic view, a single Ag-NOR/18S rDNA site in *Thalassoma noronhanum* likely represents an ancestral condition for Julidini species. *Halichoeres penrosei*, the most basal species analyzed in this genus (possibly belonging to the genus *Thalassoma*, according to Rocha et al. 2010), shows intermediate features, with a single Ag-NOR and multiple 18S rDNA sites. This indicates that multiple rDNA regions is an ancestral condition and that the rDNA dynamics is an ancient trait in *Thalassoma* and *Halichoeres* genera (Fig. 4). In fact, the multiple Ag-NORs present in *Halichoeres poeyi*, *Halichoeres radiatus* and *Halichoeres brasiliensis* and the large number of rDNA sites present in *Halichoeres* suggest that the dispersal process of these sequences precedes their diversification. The wide variation in distribution and organizational patterns of these sequences in Julidini are compatible with birth-and-death processes (Rooney and Ward 2005) acting in a stochastic evolutionary model.

In turn, the 5S rDNA sequences can present a conserved chromosomal distribution, even among phylogenetically non-related taxa (Perina et al. 2011). In most eukaryotes, these sequences are organized *in tandem* repetitions, in which the non transcribed spacers (NTS) present high interspecific variations, due to insertions/deletions, minisatellites and pseudogenes (Nelson and Honda 1985, Leah et al. 1990, Alves-Costa et al. 2008). However, although evolutionarily conserved, stochastic events may promote a great dispersal of the 5S rDNA sequences in a large number of chromosomes in some Perciformes species (Affonso and Galetti 2005, Lima-Filho et al. 2014b).

The location of 45S and 5S rDNA sites in different chromosomes is the most common condition in vertebrates (Lucchini et al. 1993, Suzuki et al. 1996, Gornung 2013), indicating independent evolution of these loci. The syntenic arrangement of these rDNA classes, as found in *Halichoeres* and *Thalassoma*, is not a common feature, although it has already been reported in some main fish orders, such as Perciformes (Ghigliotti et al. 2008, Merlo et al. 2013), Characiformes (Vicari et al. 2006, Bellafronte et al. 2009, Cioffi et al. 2009), Siluriformes (Mariotto et al. 2011, Ziemniczak et al. 2012), Anguilliformes (Deiana et al. 2006), Salmoniformes (Pendás et al. 1994), Nototheniformes (Ghigliotti et al. 2007) and Tetraodontiformes (Martinez et al. 2010). In fishes, 45S/5S rDNA arrays are phylogenetically stochastic and limited to few species of a clade (Almeida-Toledo et al. 2002), and preferentially explained by random events in the course of the evolutionary trajectory of the genome (Calado et al. 2014). Thus, the phylogenetic spread of these arrangements in the Julidini clade indicates a noteworthy evolutionary stability. In fact, although the non-syntenic organization of these rDNA classes might be interpreted as a functional advantage (Martins and Galetti 2001), the persistent 18S/5S rDNA arrays in *Thalassoma* and *Halichoeres* indicates that they are feasible and, in this case, suggesting a probable adaptive condition for this multigene organization. In addition, syntenic rDNA genes may exhibit adjacent or interspersed arrangements (Artoni et al. 2015). In Julidini, hybridization signals are apparently superimposed, suggesting the occurrence of the latter kind of organization. Further fiber-FISH analyses will allow better understanding of the organization of these arrangements.

## Final remarks

The uncommon pattern of 18S and 5S rDNA synteny presented by Julidini species indicates a shared ancestral condition, in contrast to stochastic and taxonomically restricted occurrences found in other fish groups (Drouin and Sá 1995, Calado et al. 2013). In addition to phylogenetic sharing patterns, these arrangements suggest a possible adaptive organization, given that they are all active ribosomal sites (Ag-NOR positive) in this species. The differentiated 18S/5S rDNA regions in *Halichoeres* species are particularly useful in identifying phylogenetic homeologies (pairs 5 and 15), but also sufficiently divergent to represent effective cytotaxonomic markers for this genus. Although a conserved karyotypic pattern is maintained in some Labridae species, the present data reveal a significant dynamism of the ribosomal sequences, in accordance to the moderate/high rate of chromosomal diversification in this family.

## Conflict of interest

The authors declare that they have no conflict of interest.

## Ethical approval

The experimental work fulfills all ethical guidelines regarding the handling of specimens. The collection and handling of specimens followed protocols approved by the Ethics Committee on the Use of Animals of the Federal University of Rio Grande do Norte (Process 044/2015). All authors consent to participate in the publication and are in agreement with the article content.
